# Nut Consumption and Cardiovascular Risk Factors: A Cross-Sectional Study in a Mediterranean Population

**DOI:** 10.3390/nu9121296

**Published:** 2017-11-28

**Authors:** Ajka Relja, Ana Miljković, Andrea Gelemanović, Maria Bošković, Caroline Hayward, Ozren Polašek, Ivana Kolčić

**Affiliations:** 1 School of Medicine, University of Split, 21000 Split, Croatia; arelja@mefst.hr (A.R.); ana.miljkovic@mefst.hr (A.M.); ageleman@mefst.hr (A.G.); opolasek@mefst.hr (O.P.); 2 Department of Biology, University of Zagreb Faculty of Science, 10000 Zagreb, Croatia; mariaboskovic885@gmail.com; 3 Institute of Genetics & Molecular Medicine, MRC Human Genetics Unit, University of Edinburgh, Edinburgh EH8 9YL, UK; Caroline.Hayward@igmm.ed.ac.uk

**Keywords:** nuts, food intake, obesity, hypertension, diabetes, dyslipidemia, metabolic syndrome

## Abstract

Nuts are often considered beneficial for health, yet few studies have examined determinants of their intake and the associations between nut consumption and various cardiovascular disease risk factors. The aim of this study was to identify factors associated with nut intake in a Mediterranean population, in Croatia, and to investigate the association of nut intake and various cardiovascular risk factors. Methods: Subjects from the Island of Vis, Island of Korčula and the City of Split were included in this cross-sectional study (*n* = 4416 in total; 4011 without known cardiovascular disease). Survey responses, medical records and clinically relevant measurements were utilized. Multivariate ordinal and logistic regression models were used in the analysis, adjusting for known confounding factors. Results: As low as 5% of all subjects reported daily, and 11% reported weekly, nut consumption. The characteristics associated with more frequent nut intake were female gender (Odds ratio (OR) = 1.39; 95% confidence interval (CI) 1.19–1.62), highest level of education (1.42; 1.15–1.76) and material status (1.58; 1.29–1.93), smoking abstinence (1.21; 1.04–1.42 in never-smokers and 1.22; 1.02–1.46 in ex-smokers), Mediterranean diet adherence (1.87; 1.62–2.15), and absence of central obesity (1.29; 1.09–1.53), absence of diabetes (1.30; 1.02–1.66) and metabolic syndrome (1.17; 1.01–1.36). Subjects who consumed nuts had more favorable waist-to-height (overall *p* = 0.036) and waist-to-hip ratios (0.033), lesser odds of elevated fibrinogen (*p* < 0.001 in both weekly and monthly nut consumers) and reduced high-density lipoprotein (HDL) cholesterol (*p* = 0.026), compared to non-consumers. Conclusions: It appears that frequent nut consumption is an integral part of a healthy lifestyle and better socioeconomic status. A beneficial association of nut intake with cardiovascular risk factors was confirmed in this study.

## 1. Introduction

Nuts have been identified as one of the most nutrient dense foods, rich in monounsaturated and polyunsaturated fatty acids, protein, fiber, vitamins and minerals [1]. They have also been found to contain an abundance of many other important phytochemicals, including phenolic acids, phytosterols and flavonoids, proanthocyanidins and stilbenes, phytates, sphingolipids, alkylphenols and lignans, which have antioxidative, anti-inflammatory, anti-proliferative, antiviral, chemopreventive and hypocholesterolaemic effects [2]. A small but growing body of evidence suggests that nuts are protective against cardiovascular diseases, cancer, diabetes, metabolic syndrome and hypertension [3,4,5,6]. Daily nut consumption decreased the all-cause mortality risk by 20% in large cohort studies from the United States of America (USA) [7], and a combined cohort study performed both in the USA and China [8]. This finding was also confirmed in other recent meta-analyses [9,10]. Furthermore, specific mortalities due to diabetes, respiratory diseases and infectious diseases were inversely associated with nut consumption in a meta-analysis, which included 20 cohort studies [3]. Cardiovascular risk factors, namely body weight [11], blood pressure [12,13], blood lipids [14], glucose (in diabetics) [15] and uric acid [16] were also inversely associated with nut intake. Even though nuts contain very high amounts of fat, their consumption has repeatedly been shown not to be associated with weight gain [11,17,18,19], but instead with moderate weight loss or weight stability, as reported in a large prospective study, involving 373,000 participants from 10 European countries [11]. Despite these findings, there is still a common belief that nut intake is associated with weight gain risk, even among health care professionals [20]. 

The definition of a Mediterranean diet places nuts up front and demands their intake on a daily basis, together with vegetables, fruit, olive oil and cereals [21]. Unfortunately, such intense consumption is not common, even among populations of the Mediterranean region [22,23,24]. A recent study performed in the population of Dalmatia, in the coastal region of Croatia, revealed a disappointingly low prevalence of daily nut consumption, ranging from 3% on the Island of Vis to 11% in the City of Split [25].

Previously published experimental studies on nut effects were small in size and of very short durations. Hence, limited input was available for a 2015 Cochrane review, with only five randomized control trials [17]. New results from the PREDIMED interventional study from Spain have recently become available [26,27]. This study yielded some promising and confirming results, especially for the beneficial effect of the Mediterranean diet enriched with nuts for the prevention of cardiovascular disease (CVD) events, such as myocardial infarction, stroke or CVD death [28]. However, the participants of this study allocated to the nut consuming group also received nutritional education on Mediterranean diet adherence, making it hard to disassociate the positive effects of nuts from the overall healthy eating pattern [18]. 

Despite potential health benefits, investigation into the determinants of nut consumption in the general population is very scarce in the literature [10,29], leaving a substantial gap in knowledge. Therefore, the aim of this study was to determine the factors associated with nut consumption, as well as to investigate the association between nut consumption and various cardiovascular risk factors, namely central obesity indices, dyslipidemia, elevated fibrinogen, hypertension, diabetes, elevated glycated haemoglobin (HbA1c), metabolic syndrome and gout, in a population-based sample in Dalmatia, Croatia.

## 2. Materials and Methods 

### 2.1. Study Participants and Methods

This cross-sectional study initially included 4984 subjects from the “10,001 Dalmatians” project [30]. Subjects from three Dalmatian settlements were included: the Island of Vis (*n* = 1027, sampled during 2003–2004 period), Island of Korčula (*n* = 2581, sampled during 2007 and 2012–2015 period) and the City of Split, which is the second largest city in Croatia (*n* = 1012; sampled in 2008–2009 period). A population-based sampling approach was based on generalized invitations to all island inhabitants, targeting subjects who were of age (18 or more years). The sampling scheme employed direct postal invitations, radio appearances and support from the local stakeholders (general practitioners (GPs) and local governments), yielding almost systematic responses. All respondents were informed on the study aims and goals, benefits and risks, and were asked to sign an informed consent before entering the study. The study was approved by the Ethical Board of the University of Split School of Medicine (approval number 2181-198-03-04/10-11-0008). 

### 2.2. Clinical Measurements

Each subject was offered an array of clinical measurements, which included blood and urine testing, followed by blood pressure and anthropometric measurements, electrocardiography, arterial stiffness, spirometry, heel bone density (dual-energy X-ray absorptiometry, DEXA), ophthalmological examination and other clinically relevant examinations. Subjects were also asked to fill in an extensive self-administered questionnaire, which included questions on important cardiovascular disease risk factors: demographic characteristics, medical history, socioeconomic status, smoking, alcohol consumption, physical activity, and dietary habits. Elderly people and those with disabilities were offered assistance during surveying, by a team of nine trained surveyors. 

Medical records or subjects’ responses were used to extract relevant medical history information, including previous diagnoses and the use of medications. The list included hypertension, type 2 diabetes, coronary heart disease (CHD), cerebrovascular insult (CVI), cancer, bipolar disorder, hyperlipidemia and gout. 

#### 2.2.1. Blood Pressure and Anthropometric Indices

Blood pressure was measured twice by manual mercury sphygmomanometer (calibrated weekly), at least five minutes apart, in a sitting position, after at least 10 min of rest. An average value of two measurements was taken for the analysis. Subjects were considered to have hypertension if they (a) reported a previous diagnosis of hypertension or (b) reported the use of anti-hypertensive medication or (c) had a mean systolic blood pressure ≥140 mmHg or mean diastolic pressure ≥90 mmHg [31]. Anthropometric measurements were performed using standard procedures [32], including body height, body weight, waist circumference (WC) and hip circumference (HC). Besides body mass index (BMI), we also calculated waist-to-hip ratio (WHR) and waist-to-height ratio (WHtR) as relative measures of central obesity. The cut-off value for elevated WC was ≥94 cm for men and ≥80 cm for women [32]; for elevated WHR it was ≥0.85 for women and ≥0.90 for men [32], and for WHtR it was ≥0.50 for both sexes [33].

#### 2.2.2. Biochemistry

Biochemical parameters included total cholesterol, low-density lipoprotein cholesterol (LDL), high-density lipoprotein cholesterol (HDL), triglycerides, glucose, HbA_1_c, uric acid and fibrinogen. Fasting blood samples were collected from the antecubital vein into EDTA and serum tubes and pre-processed immediately in remote study sites. They were shipped frozen (−80 °C) to 2 specialized and accredited laboratories (HRN EN ISO 15189) in Zagreb (samples collected from Vis Island and Split were analyzed in “Labor Centar” and samples from Korčula Island were analyzed in “Breyer Laboratory”). Both laboratories used the same standard methods for determining concentrations of glucose, HbA_1_c, uric acid, blood lipids and fibrinogen (measured using the Clauss method).

Subjects whose fasting glucose level exceeded 7.0 mmol/L or those who reported a previous diagnosis of type 2 diabetes were considered to be diabetic [34]. Metabolic syndrome was defined using the Joint Interim Statement (JIS) definition and the cut off values used for elevated waist circumference were ≥80 cm for women and ≥94 cm for men [35]. Subjects who had elevated uric acid (≥404 μmol/L for men and ≥338 μmol/L for women [36]) or had a record in medical history were considered positive for gout. The cut-off level for elevated cholesterol was ≥5.0 mmol/L, for LDL cholesterol it was ≥3.0 mmol/L, and for triglycerides the cut-off was ≥1.7 mmol/L [35]. HDL values of ≤1.03 mmol/L for men and ≤1.29 mmol/L for women were considered reduced HDL concentrations [35]. Information on taking medications for dyslipidemia was also taken into account. The concentration of fibrinogen was considered to be lowered if it was ≤1.5 g/L, normal if it was between 1.51–4.0 g/L or elevated if the concentration was ≥4.0 g/L [37]. 

### 2.3. Socioeconomic and Lifestyle Characteristics Associated with Cardiovascular Disease Risk

Socioeconomic status was assessed according to education level and material status. Education level was measured as years of formal schooling, and later classified into three categories (corresponding to the primary, secondary and higher education system in Croatia) as follows: lower education level (with ≤8 years of finished school), intermediate (9–12 years), and higher education (≥13 years of schooling). An assessment of material status was based on 16 validated questions forming the composite index [38], consisting of a list of items in the subject’s possession (heating system, wooden floors, video/DVD recorder, telephone, computer, two TVs, freezer, dishwasher, water supply system, flushing toilet, bathroom, library with more than 100 books, paintings or other art objects, a car, vacation house or second apartment, boat). The responses were summed and classified into four quartile categories, according to the distribution in the study population (first quartile with values ≤8, second 9–10, third 11–12, and fourth quartile with values 13–16).

Lifestyle characteristics, such as smoking, alcohol intake, and physical activity were also recorded. Smoking status was assigned as current smoker, ex-smoker (stopped more than 1 year ago) and never-smoker. Alcohol intake was measured in units per week, in order to combine all the types of alcohol a subject could have consumed during the week (beer, wine and hard liquor). In cases of consumption of ≥28 units/week for men and ≥21 units/week for women, a subject was classified as an excessive drinker [39], or a moderate drinker in cases of consuming less (1–27 units/week for men and 1–20 units/week for women), while those who did not consume any alcohol were considered non-drinkers. The level of physical activity was assessed from the survey; light activity was assigned when subject reported sitting or light physical activity during both work and leisure time. A moderate level of physical activity was assigned if a subject declared a moderate level of physical exertion in at least one part of the day, while intensive activity was assigned to all subjects who reported hard labor or other intense types of physical activity during either part of the day. 

### 2.4. Assessment of Dietary Pattern and Nut Intake 

Assessment of the diet composition was based on a 55 question survey, specifically adjusted for the population of Dalmatia [25]. Each question had six possible responses regarding the usual frequency of consumption (every day, 2–3 times a week, once a week, once a month, rarely and never). The questionnaire captured the information on olive oil and other fat intake, dairy, vegetables, fruit, nuts, potatoes, cereals (rice, pasta, bread), legumes, eggs, white and red meats, fish and sea foods, sweets, and wine (for details see [25]). Adherence to the Mediterranean diet was assessed according to the recently proposed Mediterranean Diet Serving Score (MDSS), which demands daily intake of vegetables, fruit, olive oil cereals, nuts, dairy products and wine, while other groups of food should be consumed weekly [40]. The MDSS score has a maximum of 24 points, and those foods which are considered beneficial for health and should be consumed daily contribute 3 points, while foods like red meat and sweets contribute 1 point if consumed in ≤2 servings per week [25]. Finally, we also classified all subjects on the basis of their Mediterranean diet compliance, by MDSS score—the upper quartile limit was set at 14 points (out of maximum 24), indicating good adherence to Mediterranean diet [40].

Nut consumption frequency was assessed using one question, and all types of nuts were included (tree nuts and peanuts). Subjects were classified into four categories: daily nut consumers (reported nut intake “every day”), weekly consumers (reported “2–3 times a week” nut intake), monthly (reported “once a week” or “once a month”) and those who consume nuts infrequently or never (reported “rarely” or “never”). 

### 2.5. Statistical Analysis

Due to missing values for at least one of the important characteristics needed in the analysis (anthropometric measurements, biochemistry, medical history or nut consumption), we excluded 35 subjects from the Island of Vis, 146 subjects from the Island of Korčula, and 23 subjects from the City of Split. Secondly, we removed 405 subjects, since they reported a previous diagnosis of either coronary heart disease or cerebrovascular insult. These subjects were included only in the bivariate analyses, and excluded from all the multivariate analyses of the association of nut consumption with different cardiovascular risk factors, in order to test the hypothesis only in subjects at risk of cardiovascular disease. Additionally, a sub-analysis was performed, including only elderly subjects (>65 years of age, *n* = 897), who were at an increased risk for cardiovascular diseases.

All categorical variables were described using absolute numbers and percentages, and numerical variables were described with medians and interquartile ranges (IQR), due to frequent non-normal distribution. Differences between groups for categorical variables were examined with chi-squared tests, and for numerical variables with Kruskal–Wallis tests. Post-hoc analyses were performed using the Mann–Whitney U test for numerical variables and chi-squared tests for categorical variables. 

The multivariate analysis included ordinal regression and logistic regression. Ordinal regression analysis was used to identify the characteristics associated with the ordinal dependent variables: nut consumption, BMI and fibrinogen (each variable was analysed in a separate model). Covariates in these three models included sex, age (3 age group categories: 18–34.9 years, 35.0–64.9 years, and ≥65.0 years, as in our previous study [25]), place of residence (Vis, Korčula, Split), education attainment, quartiles of material status, smoking, alcohol intake, Mediterranean diet adherence (MDSS ≥14 points), nut consumption (only in BMI and fibrinogen models), physical activity, WHtR, and four chronic diseases (hypertension, diabetes, metabolic syndrome, and gout). In all 3 models the highest value of the ordinal dependent variable was used as a referent point (daily nut consumption; BMI ≥30 kg/m^2^; fibrinogen ≥4.0 g/L). Beta values were transformed into odds ratios using the exponential value of beta, with 95% confidence interval (exponential values of beta’s lower and upper bounds).

A logistic regression analysis was used to identify the characteristics associated with the binary outcome variables: BMI, waist circumference, WHR, WHtR as binary outcomes (normal or elevated), hypertension, diabetes, elevated HbA_1_c, metabolic syndrome, gout, elevated triglycerides, elevated cholesterol, elevated LDL cholesterol, and decreased HDL cholesterol. All the logistic regression models were controlled for known confounding factors: sex, age, cohort effect (place of residence), years of schooling, quartiles of material status, smoking, alcohol intake, MDSS compliance, nut consumption, physical activity, WHtR and four chronic diseases (hypertension, diabetes, metabolic syndrome and gout), except in models where these were dependent variables. Metabolic syndrome was omitted as a covariate in the regression models for elevated triglycerides, cholesterol, LDL cholesterol, HDL cholesterol and fibrinogen, due to its failure to meet the model diagnostic criteria (Hosmer–Lemeshow test). On the other hand, models with chronic diseases as outcome variables did not include metabolic syndrome as a covariate. Using this approach, all the models built for the analysis had a good fit (Hosmer and Lemeshow test *p*-values were > 0.05). Significance level was set at *p* < 0.05 (two-sided). All statistical analyses were performed using IBM SPSS Statistics v19 (IBM, Armonk, NY, USA).

## 3. Results

This cross-sectional study included 4416 subjects from three populations, two from eastern Adriatic islands and one mainland population on the coastline. Almost all of the characteristics differed between the sub-samples according to the place of residence, except the gender composition, Mediterranean diet serving score (MDSS), and CVI in the medical history (Table 1). Overweight, obesity and some of the chronic diseases were highly prevalent, with as many as 405 subjects (9.2%) who reported having CVD in their medical history, and some of them reported having both CHD and CVI (Table 1). Daily nut consumption was reported in 7.1% of subjects from Split, 4.7% in subjects from Korčula, while only 2.7% of subjects from the Island of Vis consumed nuts daily (Table 1).

A high prevalence of metabolic syndrome was observed (as high as 60.9% in Vis), followed by hypertension (ranged from 23.9% in Split to 30.8% in Korčula), while obesity was also most common in Vis (26.2%) (Table 1).

A bivariate analysis of the entire sample (*n* = 4416) revealed that nut consumption frequency was associated with all of the investigated characteristics, except with HDL cholesterol (Table S1). Daily consumption was more common in women (75.5% vs. 24.5% in men), subjects who never smoked (54.7% vs. 19.3% in current smokers), and those compliant with the Mediterranean diet (69.3% vs. 30.7% in non-compliant), suggesting a pattern of a healthier lifestyle (Table S1). Subjects who never consumed nuts had a higher prevalence of obesity (BMI ≥ 30 kg/m^2^ in 19.9% non-consumers vs. 12.7% in daily consumers) and a higher prevalence of central obesity (elevated WHR in 74.6% vs. 64.9% in daily consumers, *p* = 0.002; elevated WHtR in 81.7% non-consumers vs. 75.4% in daily consumers, *p* = 0.024; Table S1). A similar pattern was also present in subjects who consumed nuts weekly and monthly (Table S1). There was no difference in the bivariate analysis for the prevalence of elevated cholesterol levels or LDL and HDL cholesterol levels, between daily consumers of nuts and non-consumers, while triglycerides were more frequently elevated in non-consumers (29.3% vs. 19.3% in daily consumers, *p* = 0.002; Table S1). Daily consumers and non-consumers had a similar prevalence of hypertension, diabetes, and gout, while metabolic syndrome was more frequent in non-consumers (52.3% vs. 42.6%, *p* = 0.008, Table S1).

When the confounding variables were taken into account in the multivariate ordinal regression model and only those subjects without a previous CVD diagnosis were included (*N* = 4011), several characteristics remained independently associated with nut consumption (Table 2). Higher odds for more frequent nut consumption were recorded in women (odds ratio (OR) = 1.39; 95% confidence interval (CI) 1.19–1.62; *p* < 0.001), subjects with the highest educational attainment (OR = 1.42; 95% CI 1.15–1.76; *p* = 0.001, compared to lowest), highest quartile of material status (OR = 1.58; 95% CI 1.29–1.93; *p* < 0.001, compared to lowest), non-smoking status compared to current smokers (OR = 1.21, 95% CI 1.04–1.42; *p* = 0.015 in never-smokers and OR = 1.22, 95% CI 1.02–1.46; *p* = 0.034 in ex-smokers), and compliance with the Mediterranean diet (OR = 1.87; 95% CI 1.62–2.15; *p* < 0.001) (Table 2). A similar finding was also recorded in subjects who were exposed to less intensive physical activity (OR = 1.34; 95% CI 1.04–1.73; *p* = 0.023 for light and OR = 1.30; 95% CI 1.04–1.63; *p* = 0.022 for moderate physical activity, compared to intensive), subjects who were not centrally obese (OR = 1.29; 95% CI 1.09–1.53; *p* = 0.003) and those who did not have diabetes (OR = 1.30; 95% CI 1.02–1.66; *p* = 0.031), or metabolic syndrome (OR = 1.17; 95% 1.01–1.36; *p* = 0.042) (Table 2).

Nut consumption frequency was associated with an elevated waist-to-height ratio (WHtR, overall *p* = 0.036) and waist-to-hip ratio (WHR, overall *p* = 0.033), while no association was recorded for waist circumference or BMI in subjects without a previous CVD diagnosis (Table 3). Subjects who reported weekly nut consumption had lower odds for elevated WHtR, compared to those who never consumed nuts (OR = 0.72, 95% CI 0.54–0.96; *p* = 0.026), similarly for subjects who consumed nuts monthly (OR = 0.78, 95% CI 0.63–0.97; *p* = 0.022). Daily nut consumers exhibited no difference compared to the non-consumers (OR = 0.70; 95% CI 0.45–1.08; *p* = 0.104; Table 3). A similar finding was for elevated WHR, where subjects who consumed nuts weekly and monthly had 23% lower odds for being centrally obese (OR = 0.77, 95% CI 0.60–0.99; *p* = 0.045 and OR = 0.77, 95% CI 0.64–0.93; *p* = 0.007, respectively), while daily consumers had a non-significant result (OR = 0.87; 95% CI 0.60–1.28; *p* = 0.485; Table 3). Other characteristics associated with the presence of central obesity, in both WHtR and WHR models, included male gender, older age, living in Korčula (compared to Split), lower educational attainment, ex-smoker status compared to never-smoked (only for WHtR), excessive alcohol intake (only for WHR). Higher material status (only for WHtR), current smoking, compared to never-smokers, and absence of hypertension, diabetes (only for WHR), metabolic syndrome and gout were associated with decreased odds of being centrally obese (Table 3).

Among investigated biochemical parameters, nut consumption was associated with HDL cholesterol, fibrinogen, total cholesterol and LDL cholesterol, while triglycerides did not exhibit such an association (Table 4). Subjects who consumed nuts monthly had lower odds for a decreased level of HDL cholesterol compared to non-consumers (OR = 0.75, 95% CI 0.62–0.92; *p* = 0.005), while subjects who consumed nuts weekly and daily also had the same result, but the difference did not reach statistical significance in pair-wise comparisons, even though the overall *p*-Value indicated a significant result (*p* = 0.026, Table 4). On the other hand, only subjects who consumed nuts weekly had increased odds for the concomitant presence of increased total cholesterol and LDL cholesterol, but without overall significance (OR = 1.35, 95% CI 1.02–1.79; *p* = 0.037 and OR = 1.46, 95% CI 1.11–1.94; *p* = 0.008, respectively; Table 4). Subjects who consumed nuts had lower odds for having an elevated fibrinogen level (OR = 0.66, 95% CI 0.56–0.79; *p* < 0.001 in monthly consumers, OR = 0.65, 95% CI 0.52–0.83; *p* < 0.001 in weekly consumers), while for daily consumers the result was just above the significance threshold limit, Table 4). In a sub-analysis, including only elderly subjects (>65 years of age) who are at increased risk for cardiovascular diseases, only one statistically significant association was found: between nut intake and elevated fibrinogen level (OR = 0.58, 95% CI 0.39–0.84; *p* = 0.004 in subjects consuming nuts monthly compared to non-consumers; Table S2).

When clinical outcomes were investigated for the association between nut intake and presence of chronic diseases, only one result was significant: monthly consumers had 20% lower odds for having metabolic syndrome compared to the never-consumers (OR = 0.80, 95% CI 0.66–0.96; *p* = 0.016; Table 5). Interestingly, participants who did not adhere to the Mediterranean diet had lower odds for the concomitant presence of both diabetes and elevated HbA1c (OR = 0.66, 95% CI 0.51–0.86; *p* = 0.002; OR = 0.57, 95% CI 0.43–0.75; *p* < 0.001, respectively; Table 5), which indicates that subjects with diabetes had better adherence to this preventative nutritional pattern. There was no association between nut intake and hypertension or gout presence (Table 5). In a sub-analysis, including only participants older than >65 years of age, there was only one significant association with nut intake: elevated HbA1c (OR = 0.29, 95% CI 0.10–0.87; *p* = 0.028 in daily nut consumers compared to non-consumers; Table S3).

## 4. Discussion

The results of this study indicate that as low as 5% of all subjects reported daily, and 11% reported weekly, nut consumption. More frequent nut consumption was reported by women, people with the highest level of education and material status and those with healthier lifestyles—non-smokers, more adherent to the Mediterranean diet and in subjects who were free from central obesity, diabetes and metabolic syndrome. Subjects who reported weekly and monthly nut intake were less likely to suffer from elevated waist-to-height and waist-to-hip ratios, elevated fibrinogen and reduced HDL cholesterol, compared to non-consumers.

A low prevalence of nut consumption was previously reported in Croatia—as low as 5.5% of island inhabitants reported daily nut consumption [25]. This percent varies even across the Mediterranean region, ranging from 55% of women in Southern Spain [40], to as low as 8% in the Molise region of Italy [41]. However, these figures also seem to show a positive pattern of change across time, as seen in the case of Northern Italy, where an increase in nut consumption was recorded between 2010 and 2016 [22]. A similar increase in nut consumption in the population of Southern Croatia can be seen only within the population of the City of Split, where 7% of participants consumed nuts daily in 2008–2009 period, while in 2012–2013 period, there 11% were daily consumers [25]. The same pattern was not present in the remote island populations of Vis, possibly due to less favorable socioeconomic environment and remoteness.

A recent study in New Zealand investigated predictors of nut consumption, where the daily whole nut consumption frequency of 6.9% was similar to our findings [29]. That study identified several factors associated with daily nut consumption: older age (51–70 years OR = 5.99, 95% CI 2.84–12.66), while overweight and obese subjects had reduced odds for daily whole nut intake (0.62, 0.39–0.97 in overweight, and 0.54, 0.33–0.89 in obese subjects) [29]. Additionally, the mean amount of whole nuts eaten in grams per day was associated with sex, age, ethnicity and deprivation, an area-based measure which reflected both material and social deprivation [29]. Similar results were obtained in our study, except that we did not detect the age effect on nut consumption.

Nut consumption, in this study, was associated with reduced odds for decreased HDL cholesterol and reduced odds for increased fibrinogen, while total cholesterol and LDL cholesterol did not reach the significance level (when the overall *p*-Value was observed) and triglycerides did not exhibit any association with nut intake within this study. Previous studies have also shown various results in regard to the impact of nut consumption on blood lipid levels. Some have found beneficial effects, while others did not record any difference in blood lipids between nut consumers and non-consumers [14,17,42,43,44]. A possible explanation for these findings is in the different definitions used for nut consumption (type, frequency and amount of nuts consumed), different time frames of follow-up and different confounding factors taken into account. For instance, in a Cochrane review and meta-analysis with three trials included, substantial heterogeneity was found between the trials for the effect of nut consumption on total cholesterol and triglyceride levels, while for HDL cholesterol and LDL cholesterol, no effect of nut consumption has been identified [17]. 

Subjects in this study who consumed nuts weekly and monthly had lower odds for having an elevated fibrinogen level, which is considered an inflammatory marker. This is a confirmation of a previous finding of lower levels of fibrinogen (and other inflammatory markers) in people who consumed nuts and seeds more frequently [45]. In regard to this, the finding of a recent study in the urban population of Catania, Italy, was particularly interesting because it demonstrated that the main dietary sources of total polyphenols were nuts [46]. It is therefore plausible that the cardiovascular diseases risk reduction effect of nut intake is, at least in part, due to these compounds, which are well known to counteract the burden of oxidative stress and thus limit the effects of cellular aging [47].

The nut intake effect on glucose and insulin levels and diabetes risk has also been previously investigated. The majority of studies have shown a beneficial impact of nut consumption. For example, a meta-analysis of 12 randomized controlled dietary trials, including individuals with diabetes showed that daily nut consumption (a median dose of 56 g/day over ≥3 weeks) lowered HbA1c by 0.07% (95% CI: −0.10, −0.03%; *p* = 0.0003) and fasting glucose by 0.15 mmol/L (95% CI: −0.27, −0.02 mmol/L; *p* = 0.03), compared to control diets [15]. Another meta-analysis, including 25 observational studies and two clinical trials, found that nut consumption was associated with a reduction in diabetes risk by 13% (based on six studies and 13,308 events; RR: 0.87, 95% CI 0.81–0.94) [48]. Furthermore, the PREDIMED study showed that people who consumed a Mediterranean diet enriched with daily nut intake (30 g/day; 15 g of walnuts, 7.5 g of hazelnuts, and 7.5 g of almonds) had an 18% reduction in risk for the incidence of diabetes (hazard ratio of 0.82, 95% CI 0.61–1.10) compared to the control group, who received advice on a low-fat diet [28]. Our cross-sectional study failed to confirm these results. On the contrary, subjects who did not comply with a Mediterranean diet had lower odds for the presence of diabetes and elevated HbA1c (OR = 0.66, 95% CI 0.51–0.86 and OR = 0.57, 95% CI 0.43–0.75, respectively). Since this is a cross-sectional study, this result is easily explained by the adoption of a healthy dietary pattern in those who were diagnosed with type 2 diabetes, as a tertiary prevention measure. A similar result was recorded for total and LDL cholesterol, since subjects who consumed nuts weekly presented increased odds for concomitant presence of increased levels of these blood lipids. This finding could also be a consequence of a high-fat diet content, which has been shown to raise plasma cholesterol, LDL cholesterol, and HDL cholesterol [49]. 

More frequent nut consumption in this study was also seen in subjects who were free from diabetes, metabolic syndrome and central obesity (defined by the waist-to-height and waist-to-hip ratios). At the same time, there was no association with direct measures, namely waist circumference and BMI. Interestingly, relative measures have been shown to be better for detecting cardiometabolic risk factors and adverse health outcomes [33], indirectly suggesting that nuts could be contributing to the favorable cardiovascular profile. A large population-based cohort study, with over 370,000 people, reported that those who consumed nuts >1 time/week exhibited a slightly reduced weight gain (−0.10 kg; 95% CI −0.15, −0.04) over a 5 year of follow-up period (compared to non-consumers), and had a 5% lower risk of becoming overweight or obese, compared to non-consumers [11]. A potential explanation for these findings could be found in the high satiety effect of nuts, such as almonds, whose consumption has been shown to have the same satiety impact as baked foods with the equivalent energy and macronutrient content [50]. A similar result was obtained in another experimental study in subjects at increased risk of type 2 diabetes, where almond consumption, especially eaten as a snack, lowered serum glucose responses post-prandially and managed to reduce hunger and desire to eat [51]. Another study confirmed that the metabolizable energy value of walnuts was as much as 21% smaller than predicted, possibly explaining why nut intake should not be considered a risk factor for excessive weight gain [52], especially if consumed moderately (about 30 g per day). These findings are especially important, given the trends of obesity worldwide, since nut consumption could be used in metabolism and appetite control. Systematic education about the potential health benefits of nut intake, with a special emphasis on the absence of obesity risk, for both health professionals and lay population is needed. Good quality studies and evidence based dietary guidelines should be put forward in health education and promotion, in order to clarify doubts and correct erroneous assumptions about nut intake. Indeed, an international group of authors recently published lifestyle recommendations for the prevention and management of metabolic syndrome and these included the consumption of nuts, alongside legumes, cereals (whole grains), fruits, vegetables, fish, and low-fat dairy products [53].

Habitual nut intake, as a preventive measure, could offer protection against cardiovascular disease complications and death, especially in people with lower adherence to the Mediterranean diet [14,41] and diabetes [43]. We found no such association between nut intake and the presence of the diagnosis of hypertension, diabetes or gout, but we did find some indication of the association between monthly nut intake and lower odds for metabolic syndrome presence (OR = 0.80, 95% CI 0.66–0.96). A recent meta-analysis of randomized controlled trials also identified a beneficial effect of tree nuts on metabolic syndrome criteria, through modest decreases in triglycerides and fasting blood glucose [54].

### Study Limitations and Advantages

Study limitations include cross-sectional study design, which cannot establish a causal relationship between the cause and the effect. Namely, participants who experienced a decline in their health could have started consuming nuts as a preventive measure, which could be seen in observed association between weekly nut consumption and elevated cholesterol and LDL cholesterol. Recall bias and other types of bias, like volunteer bias, due to the convenience sampling approach, and confounding by indication, could have affected the results and conclusions. The question on nut consumption included all nuts, not distinguishing between different types of nuts or the amount of consumed nuts, which disabled us from further detailed characterization of specific nut consumption. A low frequency of 5% of daily nut consumers could have affected the statistical power for detecting the association between daily nut intake and investigated risk factors. This could explain the results obtained in the regression models, without any statistically significant result for the association between daily nut intake and the outcomes compared to non-consumers, unlike for weekly and monthly nut intake groups. Additionally, this study was stretched over a 2003–2015 period, and performed as a series of cross-sectional studies, introducing the possibility for time trends in nut consumption of unknown direction to influence the results differently in the three observed populations, according to the place of residence. Each study site was sampled in different time periods, which were never over-lapping. This issue was mitigated by using the multivariate approach in the analysis, and the place of residence was one of the predictors included in the models. The future research direction that could corroborate current findings is a follow-up analysis of the nut intake frequency and the association of nut intake and health outcomes in this population. Further study should also take into account the effect of aging and the trend of nut consumption.

Interestingly, we found that a less intense physical activity level was also associated with more frequent nut consumption. This particular finding could be explained by the fact that self-reported physical activity level did not describe only leisure physical activities promoting health, like sports or gym, but an overall daily living activity, including work. The minority of subjects who reported intensive physical activity (10%) most commonly were manual workers, who usually have a lesser educational level, which in turn could be associated with decreased health awareness and reduced odds of nut consumption.

The advantage of the study is its relatively large sample size, gathered from a general population in the Mediterranean region of Croatia. Another good aspect of this study is the analysis of the association of nut intake with many health outcomes, which is seldom seen in the literature. To the best of our knowledge, this is the first study that has examined both the characteristics associated with habitual nut consumption frequency and the association between nut intake and cardiovascular health related risks in Croatia and beyond.

This study adds new insights into the existing knowledge of factors associated with nut intake and the impact of nut consumption on human health. Still, there is much to be investigated, since there are many discrepancies in results from different studies, especially con the effect of nut intake on intermediate risk factors, like blood lipid levels, and also on clinical endpoints (cardiovascular diseases, diabetes, cancer, etc.). In order to elucidate the actual mechanisms and unbiased effects of nut intake on various health outcomes, a larger size and well controlled experimental study (for many confounding factors) and of longer duration is needed. A good model could be found in the PREDIMED study, but within that study, nuts were used on top of the Mediterranean diet intervention, so it was not easy to estimate the effects of nuts themselves [28]. Nevertheless, there is a sufficient amount of evidence pointing to various health benefits of habitual nut intake, which should be included in the recommendations for prevention and management of overweight and obesity, as well as common cardiovascular risk factors.

## 5. Conclusions

The results of this study bring forward important findings, identifying several factors associated with more frequent habitual nut intake and beneficial associations between nut intake and several cardiovascular risk factors. Given the potential health benefits of nut consumption, these insights should be taken into account when planning for cardiovascular health interventions and their implementations, on both an individual and population level.

## Figures and Tables

**Table 1:** Demographic characteristics, prevalence of chronic diseases, lifestyle characteristics, Mediterranean diet compliance (MDSS ≥ 14) and nut consumption frequency according to the place of residence.

	Island of Vis *n* = 992	Island of Korčula *n* = 2435	City of Split *n* = 989	Overall *p* (Post-Hoc Test *p*-Values)
**Sex; *n* (%)**				0.050 (0.014 ^VK^, 0.158 ^VS^, 0.451 ^KS^)
Men	414 (41.7)	907 (37.2)	382 (38.6)	
Women	578 (58.3)	1528 (62.8)	607 (61.4)	
**Age (years);** median (interquartile range (IQR))	56.00 (23.0)	55.0 (23.0)	52.0 (21.0)	<0.001 (<0.001 ^VK^, <0.001 ^VS^, <0.001 ^KS^)
**Education (years of schooling);** median (IQR)	11.0 (4.0)	12.0 (3.0)	12.0 (4.0)	<0.001 (<0.001 ^VK^, <0.001 ^VS^, <0.001 ^KS^)
**Material status;** median (IQR)	10.0 (5.0)	10.0 (3.0)	12.0 (3.0)	<0.001 (<0.001 ^VK^, <0.001 ^VS^, <0.001 ^KS^)
**BMI (kg/m^2^); *n* (%)**				<0.001 (<0.001 ^VK^, 0.027 ^VS^, <0.001 ^KS^)
<25.0 (normal)	301 (30.3)	1287 (52.9)	345 (34.9)	
25.0–29.9 (overweight)	431 (43.4)	830 (34.1)	429 (43.4)	
≥30.0 (obese)	260 (26.2)	318 (13.1)	215 (21.7)	
**CVD**; *n* (%)	139 (14.0)	214 (8.79)	52 (5.3)	<0.001 (<0.001 ^VK^, <0.001 ^VS^, <0.001 ^KS^)
CHD; *n* (%)	117 (11.8)	184 (7.6)	40 (4.0)	<0.001 (<0.001 ^VK^, <0.001 ^VS^, <0.001 ^VS^)
CVI; *n* (%)	30 (3.0)	50 (2.1)	15 (1.5)	0.061 (0.088 ^VK^, 0.024 ^VS^, 0.297 ^VS^)
**Diabetes (history)**; *n* (%)	65 (6.6)	174 (7.1)	42 (4.2)	0.007 (0.536 ^VK^, 0.023 ^VS^, 0.002 ^KS^)
**Hypertension (history)**; *n* (%)	294 (29.6)	750 (30.8)	236 (23.9)	<0.001 (0.502 ^VK^, 0.004 ^VS^, <0.001 ^KS^)
**Metabolic syndrome**; *n* (%)	604 (60.9)	1048 (45.3)	353 (35.7)	<0.001 (<0.001 ^VK^, <0.001 ^VS^, <0.001 ^VS^)
**Gout (history)**; *n* (%)	63 (6.4)	157 (6.5)	28 (2.8)	<0.001 (0.912 ^VK^, <0.001 ^VS^, <0.001 ^KS^ )
**Smoking**; *n* (%)				<0.001 (<0.001 ^VK^, 0.072 ^VS^, 0.015 ^KS^)
current smoker	283 (28.5)	662 (27.2)	262 (26.5)	
ex-smoker	300 (30.2)	551 (22.6)	269 (27.2)	
never-smoker	409 (41.2)	1222 (50.2)	458 (46.3)	
**Alcohol intake**; *n* (%)				<0.001 (<0.001 ^VK^, 0.255 ^VS^, <0.001 ^KS^)
excessive	150 (15.1)	484 (19.9)	130 (13.1)	
moderate	435 (43.9)	1137 (46.7)	466 (47.1)	
none	407 (41.0)	814 (33.4)	393 (39.7)	
**Physical activity**; *n* (%)				<0.001 (<0.001 ^VK^, <0.001 ^VS^, <0.001 ^KS^)
light	257 (25.9)	481 (19.8)	351 (35.5)	
moderate	574 (57.9)	1698 (69.7)	603 (61.0)	
intensive	161 (16.2)	256 (10.5)	35 (3.5)	
**MDSS ≥ 14 points**; *n* (%)	306 (30.8)	668 (27.4)	300 (30.3)	0.068 (0.045 ^VK^, 0.804 ^VS^, 0.088 ^KS^)
**Nut consumption; *n* (%)**				<0.001 (<0.001 ^VK^, <0.001 ^VS^, <0.001 ^KS^)
infrequently or never	785 (79.1)	1162 (47.7)	494 (49.9)	
monthly	132 (13.3)	880 (36.1)	264 (26.7)	
weekly	48 (4.8)	278 (11.4)	161 (16.3)	
daily	27 (2.7)	115 (4.7)	70 (7.1)	

BMI: body mass index. CVD: cardiovascular disease. CHD: coronary heart disease. CVI: cerebrovascular insult. MDSS: Mediterranean Diet Serving Score. *p*-values for categorical variables were obtained with chi-squared tests, and for numerical variables with the Kruskal–Wallis test. Post-hoc test *p*-values for categorical variables were obtained with chi-squared tests, and for numerical variables with the Mann–Whitney U test. ^VK^ Post-hoc test *p*-Value: Vis vs. Korčula. ^VS^ Post-hoc test *p*-Value: Vis vs. Split. ^KS^ Post-hoc test *p*-Value: Korčula vs. Split.

**Table 2:** Characteristics associated with nut consumption, as determined by the ordinal regression model (sample size is 4011 subjects without a previous diagnosis of cardiovascular diseases; all independent variables included in the model are listed in the table).

	Daily Nut Consumption Adjusted Odds Ratio (95% Confidence Interval); *p*-Value
**Sex** (referent (ref): male)	
Female	1.39 (1.19–1.62); <0.001
**Age** (years, ref: 18–34.9)	
≥65.0	0.98 (0.76–1.23); 0.897
35–64.9	1.03 (0.85–1.25); 0.767
**Place of residence** (ref: Split)	
Island of Vis	0.34 (0.28–0.42); <0.001
Island of Korčula	1.12 (0.96–1.31); 0.140
**Education** (years of schooling, ref: 0–8)	
≥13	1.42 (1.15–1.76); 0.001
9–12	1.15 (0.95–1.39); 0.143
**Material status** (ref: 1st quartile)	
4th quartile	1.58 (1.29–1.93); <0.001
3rd quartile	1.17 (0.97–1.42); 0.103
2nd quartile	1.17 (0.97–1.42); 0.096
**Smoking** (ref: current smoker)	
never-smoker	1.21 (1.04–1.42); 0.015
ex-smoker	1.22 (1.02–1.46); 0.034
**Alcohol intake** (ref: none)	
excessive	1.18 (0.96–1.45); 0.123
moderate	1.15 (0.99–1.33); 0.059
**MDSS compliance** (ref: no)	
yes	1.87 (1.62–2.15); <0.001
**Physical activity** (ref: intensive)	
light	1.34 (1.04–1.73); 0.023
moderate	1.30 (1.04–1.63); 0.022
**WHtR** (≥0.5, ref: yes)	
no	1.29 (1.09–1.53); 0.003
**Hypertension** (systolic ≥140 mmHg or diastolic ≥90 mmHg or treated for hypertension, ref: yes)	
no	0.96 (0.82–1.13); 0.633
**Diabetes** (≥7 mmol/L or treated for diabetes type 2, ref: yes)	
no	1.30 (1.02–1.66); 0.031
**Metabolic syndrome** (ref: yes)	
no	1.17 (1.01–1.36); 0.042
**Gout** (uric acid ≥404 μmol/L ^♂^, ≥338 μmol/L ^♀^ or treated for gout, ref: yes)	
no	1.04 (0.87–1.25); 0.647

MDSS: Mediterranean Diet Serving Score. WHtR: waist to height ratio. ^♂^: males. ^♀^: woman. The responses for material status were summed and classified into four quartile categories, according to the distribution in the study population (first quartile with values ≤8, second 9–10, third 11–12, and fourth quartile with values 13–16). Adjusted odds ratios, 95% confidence intervals and *p*-Values were calculated using a multivariate ordinal regression model, simultaneously adjusted for all the covariates listed in this table.

**Table 3:** Association of anthropometric indices with nut consumption frequency as determined by the regression models (sample size is 4011 subjects without previous cardiovascular disease diagnosis; all independent variables included in the model are listed in the table).

	Waist-to-Height Ratio (≥0.5) Adjusted Odds Ratio (95% Confidence Interval); *p*-Value	Waist-to-Hip Ratio (≥0.90 cm ^♂^, ≥0.85 cm ^♀^) Adjusted Odds Ratio (95% Confidence Interval); *p*-Value	Waist Circumference (≥94 cm ^♂^, ≥80 cm ^♀^) Adjusted Odds Ratio (95% Confidence Interval); *p*-Value	Body Mass Index (Referent ≥ 30) Adjusted Odds Ratio (95% Confidence Interval); *p*-Value
**Sex** (referent (ref): female)				
Male	1.97 (1.57–2.46); <0.001	2.55 (2.08–3.12); <0.001	0.35 (0.28–0.43); <0.001	2.34 (2.01–2.73); <0.001
**Age** (years, ref: 18–34.9)	Overall *p* < 0.001	Overall *p* < 0.001	Overall *p* < 0.001	-
≥65	7.95 (5.34–11.85); <0.001	6.70 (4.84–9.29); <0.001	2.85 (2.03–4.01); <0.001	1.55 (1.20–2.00); 0.001
35–64.9	3.64 (2.91–4.55); <0.001	4.12 (3.28–5.17); <0.001	2.45 (1.97–3.05); <0.001	2.06 (1.68–2.54); <0.001
**Place of residence** (ref: Split)				
Island of Vis	0.87 (0.65–1.15); 0.326	1.18 (0.92–1.53); 0.195	0.67 (0.51–0.89); 0.005	0.80 (0.66–0.98); 0.029
Island of Korčula	1.41 (1.14–1.76); 0.002	1.35 (1.11–1.64); 0.003	1.10 (0.88–1.36); 0.410	0.38 (0.32–0.45); <0.001
**Education** (years of schooling, ref: ≥13)	Overall *p* < 0.001	Overall *p* < 0.001	Overall *p* < 0.001	-
0–8	2.36 (1.67–3.34); <0.001	2.42 (1.81–3.24); <0.001	2.06 (1.49–2.86); <0.001	1.23 (1.00–1.52); 0.055
9–12	1.30 (1.06–1.60); 0.013	1.21 (1.00–1.46); 0.054	1.24 (1.01–1.52); 0.041	1.16 (0.99–1.36); 0.068
**Material status** (ref: 4th quartile)	Overall *p* = 0.017	Overall *p* = 0.462	Overall *p* = 0.381	-
1st quartile	0.65 (0.49–0.87); 0.003	0.99 (0.76–1.27); 0.908	0.78 (0.59–1.04); 0.089	0.86 (0.70–1.06); 0.154
2nd quartile	0.94 (0.72–1.22); 0.639	1.17 (0.92–1.47); 0.200	0.93 (0.73–1.20); 0.601	0.88 (0.73–1.07); 0.197
3rd quartile	0.84 (0.65–1.07); 0.157	1.09 (0.87–1.36); 0.444	0.93 (0.73–1.18); 0.560	0.94 (0.78–1.13); 0.498
**Smoking** (ref: never-smoker)	Overall *p* < 0.001	Overall *p* = 0.173	Overall *p* = 0.039	-
current smoker	0.81 (0.66–1.00); 0.046	1.04 (0.86–1.26); 0.657	1.01 (0.82–1.24); 0.952	0.81 (0.69–0.95); 0.011
ex-smoker	1.36 (1.06–1.74); 0.015	1.22 (0.99–1.51); 0.064	1.33 (1.05–1.67); 0.017	1.13 (0.97–1.33); 0.127
**Alcohol intake** (ref: none)	Overall *p* = 0.454	Overall *p* = 0.028	Overall *p* = 0.865	-
excessive	0.93 (0.68–1.27); 0.663	1.46 (1.10–1.93); 0.008	1.03 (0.77–1.37); 0.849	0.83 (0.68–1.02); 0.083
moderate	0.88 (0.72–1.08); 0.212	1.14 (0.95–1.37); 0.147	0.97 (0.79–1.19); 0.735	0.72 (0.62–0.84); <0.001
**MDSS compliance** (ref: yes)				
no	0.94 (0.75–1.16); 0.547	1.18 (0.97–1.42); 0.090	0.87 (0.70–1.08); 0.201	1.23 (1.06–1.42); 0.007
yes	1.00	1.00	1.00	1.00
**Nut consumption** (ref: infrequently or never)	Overall *p* = 0.036	Overall *p* = 0.033	Overall *p* = 0.413	-
daily	0.70 (0.45–1.08); 0.104	0.87 (0.60–1.28); 0.485	0.98 (0.63–1.53); 0.941	0.85 (0.62–1.16); 0.315
weekly	0.72 (0.54–0.96); 0.026	0.77 (0.60–0.99); 0.045	0.84 (0.63–1.11); 0.222	0.92 (0.75–1.14); 0.470
monthly	0.78 (0.63–0.97); 0.022	0.77 (0.64–0.93); 0.007	0.86 (0.70–1.05); 0.147	1.02 (0.88–1.19); 0.774
**Physical activity** (ref: intensive)	Overall *p* = 0.776	Overall *p* = 0.986	Overall *p* = 0.306	-
light	1.07 (0.74–1.55); 0.702	1.03 (0.74–1.42); 0.875	1.25 (0.88–1.77); 0.205	1.17 (0.92–1.49); 0.197
moderate	1.12 (0.81–1.55); 0.505	1.02 (0.77–1.37); 0.872	1.07 (0.79–1.46); 0.645	1.01 (0.82–1.25); 0.891
**Hypertension** (systolic ≥ 140 mmHg or diastolic ≥ 90 mmHg or treated for hypertension, ref: yes)				
No	0.56 (0.42–0.74); <0.001	0.68 (0.54–0.84); <0.001	0.70 (0.54–0.90); 0.006	0.66 (0.57–0.77); <0.001
**Diabetes** (≥7 mmol/L or treated for diabetes type 2, ref: yes)				
No	0.94 (0.56–1.58); 0.815	0.52 (0.34–0.80); 0.003	0.96 (0.61–1.52); 0.862	0.76 (0.61–0.94); 0.013
**Metabolic syndrome** (ref: yes)				
No	0.14 (0.11–0.18); <0.001	0.35 (0.29–0.42); <0.001	0.09 (0.06–0.11); <0.001	0.35 (0.30–0.40); <0.001
**Gout** (uric acid ≥ 404 μmol/L ^♂^, ≥338 μmol/L ^♀^ or gout, ref: yes)				
No	0.38 (0.26–0.55); <0.001	0.45 (0.34–0.60); <0.001	0.42 (0.31–0.58); <0.001	0.59 (0.50–0.71); <0.001

MDSS: Mediterranean Diet Serving Score. ^♂^: males. ^♀^: woman. Multivariate logistic regression models were built separately for waist-to-height ratio, waist-to-hip ratio and waist circumference. A multivariate ordinal regression model was used for the analysis of the characteristics associated with Body Mass Index (BMI). Adjusted odds ratios, 95% confidence intervals and *p*-Values were calculated using multivariate regression models; each of the four models was simultaneously adjusted for all covariates listed in this table.

**Table 4:** Characteristics associated with unfavorable biochemical parameters; lipid levels as determined by the multivariate logistic regression models and elevated fibrinogen as determined by ordinal regression model (sample size is 4011 subjects without previous cardiovascular disease diagnosis; all independent variables included in the model are listed in the table).

	Cholesterol (≥5 mmol/L) Adjusted Odds Ratio (95% Confidence Interval); *p*-Value	LDL (≥3 mmol/L) Adjusted Odds Ratio (95% Confidence Interval); *p*-Value	HDL (≤1.03 mmol/L ^♂^, ≤1.29 mmol/L ^♀^) Adjusted Odds Ratio (95% Confidence Interval); *p*-Value	Triglycerides (≥1.7 mmol/L) Adjusted Odds Ratio (95% Confidence Interval); *p*-Value	Fibrinogen (ref: ≥4.0 g/L) Adjusted Odds Ratio (95% Confidence Interval); *p*-Value
**Sex** (referent (ref): female)					
Male	0.86 (0.71–1.04); 0.128	0.92 (0.76–1.11); 0.364	0.78 (0.63–0.96); 0.020	1.95 (1.62–2.34); <0.001	0.75 (0.63–0.89); 0.001
**Age** (years, ref: 18–34.9)	Overall *p* < 0.001	Overall *p* <0.001	Overall *p* < 0.001	Overall *p* < 0.001	-
≥65	4.88 (3.59–6.64); <0.001	4.40 (3.26–5.94); <0.001	0.45 (0.32–0.63); <0.001	1.14 (0.81–1.61); 0.446	1.86 (1.39–2.48); <0.001
35–64.9	4.56 (3.67–5.66); <0.001	3.67 (2.96–4.55); <0.001	0.54 (0.42–0.70); <0.001	1.61 (1.20–2.15); 0.001	1.46 (1.16–1.84); 0.001
**Place of residence** (ref: Split)					
Island of Vis	0.97 (0.75–1.26); 0.837	0.71 (0.55–0.91); 0.007	0.10 (0.07–0.14); <0.001	0.68 (0.54–0.87); 0.002	0.51 (0.41–0.63); <0.001
Island of Korčula	0.91 (0.74–1.12); 0.369	0.89 (0.72–1.09); 0.265	0.44 (0.36–0.54); <0.001	0.65 (0.53–0.8); <0.001	0.31 (0.26–0.37); <0.001
**Education** (years of schooling, ref: ≥13)	Overall *p* = 0.595	Overall *p* = 0.117	Overall *p* = 0.703	Overall *p* = 0.392	-
0–8	1.00 (0.76–1.32); 0.995	0.89 (0.68–1.16); 0.395	1.00 (0.74–1.34); 0.989	1.19 (0.92–1.55); 0.177	1.25 (0.98–1.58); 0.069
9–12	1.09 (0.89–1.33); 0.388	1.12 (0.92–1.36); 0.259	1.08 (0.87–1.33); 0.482	1.06 (0.87–1.30); 0.539	1.25 (1.05–1.50); 0.014
**Material status** (ref: 4th quartile)	Overall *p* = 0.495	Overall *p* = 0.775	Overall *p* = 0.010	Overall *p* = 0.240	-
1st quartile	1.17 (0.90–1.52); 0.244	1.07 (0.83–1.37); 0.625	0.85 (0.64–1.12); 0.246	1.22 (0.94–1.57); 0.128	0.80 (0.64–1.00); 0.054
2nd quartile	0.98 (0.77–1.24); 0.842	0.98 (0.77–1.23); 0.843	1.02 (0.80–1.31); 0.873	1.23 (0.98–1.56); 0.078	0.73 (0.60–0.90); 0.003
3rd quartile	1.06 (0.84–1.33); 0.649	1.08 (0.86–1.36); 0.506	1.29 (1.02–1.63); 0.034	1.23 (0.98–1.54); 0.068	0.80 (0.65–0.97); 0.025
**Smoking** (ref: never-smoker)	Overall *p* = 0.311	Overall *p* = 0.364	Overall *p* < 0.001	Overall *p* < 0.001	-
current smoker	1.13 (0.93–1.38); 0.215	1.15 (0.95–1.40); 0.158	1.63 (1.32–2.00); <0.001	1.69 (1.39–2.06); <0.001	1.11 (0.93–1.32); 0.244
ex-smoker	1.15 (0.93–1.43); 0.203	1.07 (0.87–1.32); 0.505	1.07 (0.85–1.34); 0.562	1.30 (1.07–1.58); 0.009	1.03 (0.86–1.23); 0.746
**Alcohol intake** (ref: none)	Overall *p* = 0.071	Overall *p* = 0.838	Overall *p* < 0.001	Overall *p* = 0.250	-
excessive	1.38 (1.05–1.82); 0.022	1.05 (0.81–1.37); 0.703	0.49 (0.37–0.65); <0.001	1.04 (0.81–1.34); 0.744	0.58 (0.46–0.74); <0.001
moderate	1.10 (0.91–1.32); 0.344	0.98 (0.82–1.18); 0.832	0.63 (0.52–0.76); <0.001	0.89 (0.74–1.07); 0.227	0.82 (0.70–0.96); 0.015
**MDSS compliance** (ref: yes)					
no	0.94 (0.77–1.15); 0.543	1.01 (0.84–1.23); 0.881	1.18 (0.96–1.45); 0.112	0.93 (0.78–1.12); 0.437	0.78 (0.66–0.91); 0.002
**Nut consumption** (ref: infrequently or never)	Overall *p* = 0.169	Overall *p* = 0.063	Overall *p* = 0.026	Overall *p* = 0.494	-
daily	1.26 (0.83–1.92); 0.281	1.11 (0.75–1.65); 0.599	0.74 (0.48–1.13); 0.160	0.78 (0.52–1.16); 0.221	0.71 (0.51–1.01); 0.051
weekly	1.35 (1.02–1.79); 0.037	1.46 (1.11–1.94); 0.008	0.81 (0.62–1.07); 0.141	0.86 (0.65–1.13); 0.270	0.65 (0.52–0.83); <0.001
monthly	1.05 (0.86–1.27); 0.648	1.03 (0.85–1.24); 0.789	0.75 (0.62–0.92); 0.005	0.95 (0.79–1.15); 0.619	0.66 (0.56–0.79); <0.001
**Physical activity** (ref: intensive)	Overall *p* = 0.766	Overall *p* = 0.632	Overall *p* = 0.053	Overall *p* = 0.182	-
light	0.89 (0.65–1.23); 0.487	1.01 (0.75–1.36); 0.954	1.02 (0.73–1.42); 0.914	1.13 (0.85–1.51); 0.395	1.05 (0.80–1.37); 0.713
moderate	0.94 (0.71–1.25); 0.678	1.09 (0.84–1.42); 0.514	0.81 (0.60–1.10); 0.178	0.95 (0.74–1.22); 0.685	0.91 (0.72–1.15); 0.437
**WHtR** (≥0.5, ref: yes)					
no	0.53 (0.43–0.65); <0.001	0.47 (0.38–0.57); <0.001	0.41 (0.32–0.52); <0.001	0.29 (0.22–0.38); <0.001	0.74 (0.61–0.90); 0.002
**Hypertension** (systolic ≥ 140 mmHg or diastolic ≥ 90 mmHg or treated for hypertension, ref: yes)					
no	0.90 (0.72–1.11); 0.304	0.91 (0.74–1.11); 0.357	0.80 (0.66–0.99); 0.036	0.68 (0.57–0.81); <0.001	0.89 (0.75–1.05); 0.161
**Diabetes** (≥7 mmol/L or treated for diabetes type 2, ref: yes)					
no	1.48 (1.11–1.98); 0.007	1.54 (1.17–2.03); 0.002	0.56 (0.42–0.74); <0.001	0.57 (0.45–0.73); <0.001	0.86 (0.67–1.10); 0.221
**Gout** (uric acid ≥404 μmol/L ^♂^, ≥338 μmol/L ^♀^ or gout, ref: yes)					
no	0.80 (0.63–1.03); 0.083	1.04 (0.83–1.30); 0.740	0.63 (0.50–0.80); <0.001	0.41 (0.34–0.50); <0.001	1.01 (0.83–1.23); 0.895

MDSS—Mediterranean Diet Serving Score. WHtR—waist-to-height ratio. ^♂^: males. ^♀^: woman. Multivariate logistic regression models were built separately for cholesterol, LDL, HDL and triglycerides. Multivariate ordinal regression model was used for the analysis of the characteristics associated with elevated fibrinogen. Adjusted odds ratios, 95% confidence intervals and *p*-Values were calculated using multivariate regression models; each of the five models presented here was simultaneously adjusted for all covariates listed in this table.

**Table 5:** Characteristics associated with hypertension, diabetes, metabolic syndrome and gout as determined by the multivariate logistic regression analyses (sample size is 4011 subjects without previous cardiovascular disease diagnosis; all independent variables included in the model are listed in the table).

	Hypertension (Systolic ≥140 mmHg or Diastolic ≥90 mmHg or Treated for Hypertension) Adjusted Odds Ratio (95% Confidence Interval); *p*-Value	Diabetes (≥7 mmol/L or Treated for Diabetes Type 2) Adjusted Odds Ratio (95% Confidence Interval); *p*-Value	Elevated HbA1c (≥6.5 mmol/L or Treated for Diabetes Type 2) Adjusted Odds Ratio (95% Confidence Interval); *p*-Value	Metabolic Syndrome Adjusted Odds Ratio (95% Confidence Interval); *p*-Value	Gout (Uric Acid ≥404 mmol/L ^♂^-≥338 mmol/L ^♀^ or Gout) Adjusted Odds Ratio (95% Confidence Interval); *p*-Value
**Sex** (referent (ref): female)					
male	1.29 (1.06–1.56); 0.011	2.32 (1.74–3.09); <0.001	1.77 (1.29–2.42); <0.001	0.71 (0.59–0.86); <0.001	1.91 (1.55–2.37); <0.001
**Age** (years, ref: 18–34.9)	Overall *p* < 0.001	Overall *p* = 0.002	Overall *p* = 0.006	Overall *p* < 0.001	Overall *p* = 0.070
≥65	10.75 (6.66–17.36); <0.001	3.69 (1.69–8.03); 0.001	3.43 (1.42–8.31); 0.006	3.16 (2.24–4.47); <0.001	1.24 (0.83–1.86); 0.294
35–64.9	5.37 (3.41–8.46); <0.001	2.71 (1.28–5.73); 0.009	2.46 (1.04–5.79); 0.040	2.24 (1.66–3.02); <0.001	0.96 (0.67–1.38); 0.833
**Place of residence** (ref: Split)					
Island of Vis	0.62 (0.48–0.80); <0.001	0.97 (0.65–1.44); 0.876	0.96 (0.61–1.50); 0.844	2.19 (1.71–2.80); <0.001	1.44 (1.07–1.93); 0.015
Island of Korčula	0.98 (0.80–1.22); 0.888	1.25 (0.88–1.77); 0.206	1.64 (1.11–2.43); 0.013	1.09 (0.89–1.34); 0.392	1.19 (0.92–1.53); 0.186
**Education** (years of schooling, ref: ≥13)	Overall *p* = 0.001	Overall *p* = 0.087	Overall *p* = 0.089	Overall *p* = 0.002	Overall *p* = 0.006
0–8	1.44 (1.11–1.86); 0.006	1.52 (1.03–2.24); 0.035	1.51 (0.99–2.3); 0.056	1.44 (1.11–1.86); 0.006	1.62 (1.21–2.18); 0.001
9–12	0.93 (0.75–1.15); 0.494	1.17 (0.84–1.65); 0.352	1.11 (0.76–1.61); 0.599	0.98 (0.81–1.20); 0.866	1.24 (0.97–1.58); 0.086
**Material status** (ref: 4th quartile)	Overall *p* = 0.062	Overall *p* = 0.381	Overall *p* = 0.062	Overall *p* = 0.957	Overall *p* = 0.372
1st quartile	1.33 (1.03–1.71); 0.029	1.31 (0.88–1.94); 0.178	1.67 (1.07–2.60); 0.025	0.96 (0.75–1.24); 0.770	0.81 (0.61–1.08); 0.154
2nd quartile	1.25 (0.98–1.58); 0.070	1.37 (0.94–2.00); 0.100	1.63 (1.06–2.52); 0.027	0.94 (0.74–1.18); 0.573	0.79 (0.61–1.04); 0.094
3rd quartile	1.03 (0.82–1.30); 0.802	1.33 (0.91–1.92); 0.137	1.75 (1.15–2.68); 0.010	0.97 (0.78–1.21); 0.780	0.87 (0.67–1.13); 0.306
**Smoking** (ref: never-smoker)	Overall *p* = 0.001	Overall *p* = 0.706	Overall *p* = 0.698	Overall *p* = 0.897	Overall *p* < 0.001
current smoker	0.65 (0.53–0.80); <0.001	0.88 (0.63–1.21); 0.418	0.86 (0.60–1.22); 0.398	1.04 (0.86–1.27); 0.664	0.67 (0.52–0.86); 0.002
ex-smoker	0.78 (0.64–0.95); 0.014	0.93 (0.70–1.25); 0.647	0.97 (0.70–1.33); 0.832	1.03 (0.85–1.26); 0.747	1.27 (1.02–1.57); 0.031
**Alcohol intake** (ref: none)	Overall *p* = 0.969	Overall *p* = 0.062	Overall *p* = 0.005	Overall *p* = 0.057	Overall *p* = 0.005
excessive	0.99 (0.76–1.28); 0.921	0.81 (0.56–1.18); 0.274	0.69 (0.46–1.04); 0.076	1.14 (0.88–1.47); 0.312	1.60 (1.21–2.13); 0.001
moderate	1.01 (0.84–1.23); 0.882	0.71 (0.54–0.94); 0.019	0.60 (0.44–0.82); 0.001	0.88 (0.73–1.06); 0.170	1.27 (1.01–1.58); 0.038
**MDSS compliance** (ref: yes)					
no	1.14 (0.95–1.37); 0.152	0.66 (0.51–0.86); 0.002	0.57 (0.43–0.75); <0.001	0.99 (0.83–1.19); 0.932	1.15 (0.93–1.42); 0.194
**Nut consumption** (ref: infrequently or never)	Overall *p* = 0.248	Overall *p* = 0.212	Overall *p* = 0.355	Overall *p* = 0.103	Overall *p* = 0.435
daily	1.15 (0.79–1.68); 0.470	0.58 (0.31–1.08); 0.088	0.67 (0.35–1.26); 0.210	0.83 (0.57–1.21); 0.338	1.13 (0.73–1.76); 0.577
weekly	0.91 (0.69–1.20); 0.516	0.77 (0.50–1.18); 0.233	0.85 (0.54–1.34); 0.475	0.88 (0.68–1.14); 0.339	0.97 (0.71–1.33); 0.868
monthly	1.17 (0.97–1.41); 0.109	0.84 (0.63–1.12); 0.247	0.80 (0.58–1.09); 0.155	0.80 (0.66–0.96); 0.016	0.85 (0.68–1.06); 0.150
**Physical activity** (ref: intensive)	Overall *p* = 0.518	Overall *p* = 0.063	Overall *p* = 0.009	Overall *p* = 0.046	Overall *p* = 0.275
light	0.87 (0.65–1.17); 0.355	1.66 (1.08–2.55); 0.021	2.15 (1.31–3.52); 0.002	0.98 (0.73–1.32); 0.909	1.31 (0.94–1.82); 0.114
moderate	0.86 (0.66–1.11); 0.253	1.34 (0.91–1.97); 0.135	1.64 (1.04–2.57); 0.032	0.80 (0.62–1.04); 0.093	1.23 (0.92–1.64); 0.165
**WHtR** (≥0.5, ref: yes)					
no	0.52 (0.39–0.68); <0.001	1.00 (0.6–1.66); 0.986	0.57 (0.30–1.08); 0.083	0.14 (0.10–0.18); <0.001	0.39 (0.27–0.57); <0.001
**Hypertension** (systolic ≥140 mmHg or diastolic ≥90 mmHg or treated for hypertension, ref: yes)					
no	na	0.56 (0.43–0.72); <0.001	0.58 (0.44–0.77); <0.001	0.33 (0.28–0.40); <0.001	0.50 (0.41–0.61); <0.001
**Diabetes** (≥7 mmol/L or treated for diabetes type 2, ref: yes)					
no	0.57 (0.44–0.74); <0.001	na	na	0.19 (0.14–0.27); <0.001	0.91 (0.69–1.19); 0.488
**Metabolic syndrome** (ref: yes)					
no	0.33 (0.28–0.40); <0.001	0.18 (0.13–0.26); <0.001	0.25 (0.18–0.36); <0.001	na	0.61 (0.49–0.75); <0.001
**Gout** (Uric acid ≥404 μmol/L ^♂^, ≥338 μmol/L ^♀^ or gout, ref: yes)					
no	0.50 (0.41–0.61); <0.001	0.90 (0.69–1.18); 0.453	0.86 (0.64–1.15); 0.306	0.61 (0.49–0.75); <0.001	na

MDSS: Mediterranean Diet Serving Score. WHtR: waist-to-height ratio. Na: not applicable. ^♂^: males. ^♀^: woman. Multivariate logistic regression models were built separately for hypertension, diabetes, metabolic syndrome and gout. Adjusted odds ratios, 95% confidence intervals and *p*-Values were calculated using multivariate logistic regression; each of the four models was simultaneously adjusted for all covariates listed in this table, with an exception of excluding predictor variables in models where those variables were the outcome variables (marked with “na”).

## Supplementary Materials

The following are available online at www.mdpi.com/2072-6643/9/12/1296/s1.
