# Supplementary material for: Nut Consumption and Cardiovascular Risk Factors: A Cross-Sectional Study in a Mediterranean Population
Source: Nutrients. 2017 Nov 28;9(12):1296. doi: 10.3390/nu9121296 (PMC5748747; doi:10.3390/nu9121296)
Supplement: Supplementary file 1 [file nutrients-09-01296-s001.pdf]

Table S1. Characteristics associated with frequency of nut consumption (full entire sample; Nn=4,416).

	Daily nut consumption Nn= 212	Weekly nut consumption Nn= 487	Monthly nut consumption Nn= 1,276	Infrequent or never nut consumption Nn= 2,441	Overall *pP* (post-hoc test p-VP values)
**Sex; n (%)**					<0.001 (0.173^{DW}, <0.001^{DM}, <0.001^{D}[illegible] 0.001^{WM}, <0.001^{WN}, 0.054^{MN})
men	52 (24.5)	144 (29.6)	490 (38.6)	1017 (41.7)	
women	160 (75.5)	343 (70.4)	786 (61.6)	1424 (58.3)	
**Age (years);** median (interquartile range (, IQR))	58.0 (19.75)	53.0 (21.0)	51.0 (22.0)	56.0 (23.0)	<0.001 (<0.001^{DW}, <0.001^{DM}, 0.060^{D}[illegible] 0.035^{WM}, <0.001^{WN}, <0.001^{MN})
**Education (years of schooling);** median (IQR)	12.0 (4.0)	12.0 (3.0)	12.0 (3.0)	12.0 (4.0)	<0.001 (0.438 ^{DW}, 0.173^{DM}, <0.001^{DN}[illegible] 0.001^{WM}, <0.001^{WN}, <0.001^{MN})
**Material status**; median (IQR)	11.0 (4.0)	11.0 (4.0)	11.0 (4.0)	10.0 (4.0)	<0.001 (0.073^{DW}, 0.786^{DM}, <0.001^{DN}, 0.001^{WM}, <0.001^{WN}, <0.001^{MN})
**Smoking**; n (%)					0.008 (0.431^{DW}, 0.021^{DM}, 0.011^{DN}, 0.059^{WM}, 0.012^{WN}, 0.625^{MN})
current smokers	41 (19.3)	115 (23.6)	361 (28.3)	690 (28.3)	
ex-smokers	55 (25.9)	114 (23.4)	315 (24.7)	636 (26.1)	
never-smokers	116 (54.7)	258 (53.0)	600 (47.0)	1115 (45.7)	
**Alcohol intake**; n (%)					0.001 (0.098^{DW}, 0.025^{DM}, 0.403^{DN}, 0.256^{WM}, 0.012^{WN}, 0.002^{MN})
excessive	32 (15.1)	70 (14.4)	225 (17.6)	437 (17.9)	
moderate	90 (42.5)	248 (50.9)	631 (49.5)	1069 (43.8)	
none	90 (42.5)	169 (34.7)	420 (32.9)	935 (38.3)	
**MDSS≥14 points**; n (%)	147 (69.3)	189 (38.8)	301 (23.6)	637 (26.1)	<0.001 (<0.001^{DW}, <0.001^{DM}, <0.001^{DN}, <0.001^{WM}, <0.001^{WN}, 0.095^{MN})
**Physical activity**; n (%)					<0.001 (0.588^{DW}, 0.414^{DM}, 0.041^{DN}, 0.006^{WM}, <0.001^{WN}, 0.019^{MN})
light	51 (24.1)	122 (25.1)	304 (23.8)	612 (25.1)	
moderate	147 (69.3)	342 (70.2)	852 (66.8)	1532 (62.8)	
intensive	14 (6.6)	23 (4.7)	120 (9.4)	295 (12.1)	
**BMI (kg/m^2);** n (%)					<0.001 (0.550^{DW}, 0.405^{DM}, 0.017^{DN}, 0.662^{WM}, <0.001^{WN}, <0.001^{MN})
≤25.0 (normal)	102 (48.1)	242 (49.7)	605 (47.4)	984 (40.3)	
25.0-29.9 (overweight)	83 (39.2)	172 (35.3)	464 (36.4)	971 (39.8)	
≥30.0 (obese)	27 (12.7)	73 (15.0)	207 (16.2)	486 (19.9)	
**Waist circumference** (≥94 cm♂, ≥80cm♀); n (%)	169 (80.1)	360 (74.2)	919 (72.5)	1909 (78.9)	<0.001 (0.096^{DW}, 0.021^{DM}, 0.695^{DN}, 0.475^{WM}, 0.022^{WN}, <0.001^{MN})
**WHR** (≥0.90 ♂, ≥0.85♀); n (%)	137 (64.9)	296 (61.0)	805 (63.6)	1800 (74.6)	<0.001 (0.330^{DW}, 0.718^{DM}, 0.002^{DN}, 0.313^{WM}, <0.001^{WN}, <0.001^{MN})

**WHtR** (≥0.5); n %	159 (75.4)	348 (71.8)	932 (73.6)	1975 (81.7)	<0.001 (0.326^{DW}, 0.583^{DM}, 0.024^{DN}, 0.446^{WM}, <0.001^{WN}, <0.001^{MN})
**Cholesterol** (≥5 mmol/L); n (%)	164 (77.4)	378 (77.6)	892 (69.9)	1841 (75.4)	<0.001 (0.940^{DW}, 0.027^{DM}, 0.529^{DN}, 0.001^{WM}, 0.301^{WN}, <0.001^{MN})
**HDL** (≤1.03 mmol/L♂, ≤1.29 mmol/L♀); n (%)	35 (16.5)	95 (19.5)	231 (18.1)	493 (20.2)	0.321 (0.349^{DW}, 0.575^{DM}, 0.197^{DN}, 0.497^{WM}, 0.729^{WN}, 0.126^{MN})
**LDL** (≥3 mmol/L); n (%)	158 (74.5)	377 (77.4)	884 (69.3)	1795 (73.5)	0.003 (0.408^{DW}, 0.122^{DM}, 0.753^{DN}, 0.001^{WM}, 0.074^{WN}, 0.006^{MN})
**Triglycerides** (≥1.7 mmol/L); n (%)	41 (19.3)	106 (21.8)	302 (23.7)	714 (29.3)	<0.001 (0.469^{DW}, 0.166^{DM}, 0.002^{DN}, 0.397^{WM}, 0.001^{WN}, <0.001^{MN})
**CHD;** n (%)	13 (6.1)	25 (5.1)	59 (4.6)	244 (10.0)	<0.001 (0.592^{DW}, 0.346^{DM}, 0.068^{DN}, 0.658^{WM}, 0.001^{WN}, <0.001^{MN})
**CVI;** n (%)	4 (1.9)	8 (1.6)	17 (1.3)	66 (2.7)	0.041 (0.819^{DW}, 0.526^{DM}, 0.477^{DN}, 0.662^{WM}, 0.173^{WN}, 0.007^{MN})
**Hypertension** (Systolic ≥140 mmHg or Diastolic ≥90 mmHg or treated for hypertension); n (%)	73 (34.4)	141 (29.0)	391 (30.7)	896 (36.8)	<0.001 (0.148^{DW}, 0.279^{DM}, 0.496^{DN}, 0.471^{WM}, 0.001^{WN}, <0.001^{MN})
**Diabetes** (≥7 mmol/L or treated for diabetes type 2); n (%)	20 (9.4)	39 (8.0)	105 (8.2)	318 (13.0)	<0.001 (0.533^{DW}, 0.558^{DM}, 0.132^{DN}, 0.880^{WM}, 0.002^{WN}, <0.001^{MN})
**HbA1c** (≥6.5 mmol/L or treated for diabetes); n (%)	19 (9.0)	35 (7.2)	85 (6.7)	255 (10.4)	0.001 (0.419^{DW}, 0.224^{DM}, 0.496^{DN}, 0.695^{WM}, 0.028^{WN}, <0.001^{MN})
**Metabolic syndrome;** n (%)	87 (42.6)	189 (39.5)	471 (39.0)	1258 (52.3)	<0.001 (0.449^{DW}, 0.327^{DM}, 0.008^{DN}, 0.845^{WM}, <0.001^{WN}, <0.001^{MN})
**Gout** (Uric acid ≥404 µmol/L♂, ≥338 µmol/L♀ or gout); n (%)	34 (16.9)	71 (15.0)	191 (16.0)	513 (21.6)	<0.001 (0.526^{DW}, 0.747^{DM}, 0.122^{DN}, 0.602^{WM}, 0.001^{WN}, <0.001^{MN})

MDSS - Mediterranean Diet Serving Score. BMI - body mass index. WHR – waist-to-hip ratio. WHtR – waist-to-height ratio. CHD - coronary heart disease. CVI - cerebrovascular insult. ♂: males. ♀: woman. *p*-Values for categorical variables were obtained with chi-squared test, and for numerical with Kruskal-Wallis test. Post-hoc test ~~P~~ *p*-Values ~~values~~ for categorical variables were obtained with chi-squared test, and for numerical with Mann-Whitney U test. ^{DW} Post-hoc test ~~P~~*p*-V~~-v~~alue: ~~Daily~~ daily vs. ~~Weekly~~weekly.

^{DM} Post-hoc test *p*-Value~~P value~~: ~~Daily~~ daily vs. ~~Monthly~~monthly.

^{DN} Post-hoc test *p*-Value~~P value~~: ~~Daily~~ daily vs. ~~Never~~never.

^{WM} Post-hoc test *p*-Value~~P value~~: ~~Weekly~~ weekly vs. ~~Monthly~~monthly.

^{WN} Post-hoc test *p*-Value~~P value~~: ~~Weekly~~ weekly vs. ~~Infrequently~~infrequently or never.

^{MN} Post-hoc test *p*-Value~~P value~~: ~~Monthly~~ monthly vs. ~~Infrequently~~infrequently or never.

Formatted: Font: 9 pt, Not Italic, Superscript

Formatted: Font: 9 pt, Not Italic, Superscript

Formatted: Font: 9 pt, Not Italic, Superscript

Formatted: Font: 9 pt, Not Italic, Superscript

Formatted: Font: 9 pt, Not Italic, Superscript

Formatted: Font: 9 pt, Not Italic, Superscript

Table S2. Characteristics associated with unfavorable biochemical parameters; lipid levels as determined by the multivariate logistic regression models and elevated fibrinogen as determined by ordinal regression model (sample size is 897 ~~participants~~ subjects without previous cardiovascular disease~~CVD~~ diagnosis and older than 65 years of age; all independent variables included in the model are listed in the table).

	**Cholesterol (≥5 mmol/L)** Adjusted odds ratio (95% confidence interval); ~~P~~*p*-Value	**LDL (≥3 mmol/L)** Adjusted odds ratio (95% confidence interval); *p*-Value~~P~~	**HDL (≤1.03 mmol/L♂, ≤1.29 mmol/L♀)** Adjusted odds ratio (95% confidence interval); *p*-Value~~P~~	**Triglycerides (≥1.7 mmol/L)** Adjusted odds ratio (95% confidence interval); *p*-Value~~P~~	**Fibrinogen (≥4.0 g/l is referent)** Adjusted odds ratio (95% confidence interval); *p*-Value~~P~~
**Sex** (referent ~~(~~ref~~)~~: female)					
male	0.67 (0.43-1.01); 0.120	0.81 (0.52-1.27); 0.360	0.35 (0.21-0.59); <0.001	1.04 (0.71-1.53); 0.845	0.96 (0.67-1.37); 0.806
**Age (years)** median (interquartile range (IQR))	0.96 (0.92-1.00); 0.047	0.98 (0.95-1.02); 0.324	1.00 (0.96-1.05); 0.837	0.97 (0.94-1.00); 0.086	1.01 (0.98-1.05); 0.384
**Place of residence** (ref: Split)					
Island of Vis	2.07 (1.05-4.10); 0.035	1.34 (0.70-2.55); 0.379	0.09 (0.04-0.18); <0.001	0.56 (0.34-0.93); 0.024	0.41 (0.26-0.67); <0.001
Island of Korčula	1.20 (0.67-2.14); 0.538	1.01 (0.58-1.78); 0.962	0.35 (0.21-0.58); <0.001	0.44 (0.28-0.70); <0.001	0.23 (0.15-0.35); <0.001
**Education** (years of schooling, ref: ≥13)	Overall ~~P~~*p* = 0.154	Overall *p* = ~~P=~~0.043	Overall *p* = ~~P=~~0.294	Overall *p* = ~~P=~~0.328	-
0-8	0.58 (0.31-1.10); 0.101	0.59 (0.32-1.08); 0.086	1.27 (0.66-2.46); 0.471	1.46 (0.88-2.42); 0.147	2.12 (1.30-3.44); 0.002
9-12	0.88 (0.48-1.64); 0.692	1.05 (0.59-1.89); 0.864	1.60 (0.86-2.96); 0.135	1.21 (0.74-1.98); 0.448	1.49 (0.94-2.36); 0.092
**Material status** (ref: 4th quartile)	Overall *p* = ~~P=~~0.624	Overall *p* = ~~P=~~0.055	Overall *p* = ~~P=~~0.107	Overall *p* = ~~P=~~0.329	-
1st quartile	1.47 (0.77-2.80); 0.243	1.54 (0.84-2.81); 0.159	1.01 (0.51-2.00); 0.976	1.31 (0.77-2.23); 0.326	0.76 (0.46-1.24); 0.267
2nd quartile	1.32 (0.69-2.53); 0.402	1.35 (0.74-2.47); 0.334	1.12 (0.56-2.21); 0.750	1.10 (0.64-1.91); 0.726	0.88 (0.54-1.45); 0.618
3rd quartile	1.12 (0.57-2.18); 0.749	1.24 (0.65-2.34); 0.512	1.88 (0.94-3.78); 0.075	1.58 (0.90-2.77); 0.114	0.95 (0.56-1.61); 0.847
**Smoking** (ref: never-smokers)	Overall *p* = ~~P=~~0.803	Overall *p* = ~~P=~~0.408	Overall *p* = ~~P=~~0.806	Overall *p* = ~~P=~~0.226	-
current smokers	0.94(0.47-1.87); 0.860	1.44 (0.72-2.90); 0.303	0.87 (0.43-1.78); 0.712	1.56 (0.94-2.57); 0.085	1.27 (0.78-2.05); 0.340
ex-smokers	0.85 (0.52-1.38); 0.507	0.87 (0.55-1.36); 0.535	0.85 (0.50-1.45); 0.548	1.11 (0.75-1.63); 0.609	0.86 (0.59-1.24); 0.418
**Alcohol intake** (ref: none)	Overall *p* = ~~P=~~0.196	Overall *p* = ~~P=~~0.504	Overall *p* = ~~P=~~0.182	Overall *p* = ~~P=~~0.320	-
excessive	1.74 (0.87-3.48); 0.114	1.23 (0.65-2.35); 0.528	0.62 (0.30-1.26); 0.183	1.13 (0.67-1.91); 0.643	0.69 (0.41-1.15); 0.153
moderate	1.01 (0.64-1.61); 0.960	0.89 (0.57-1.38); 0.609	0.68 (0.44-1.06); 0.088	0.83 (0.57-1.20); 0.323	0.92 (0.65-1.29); 0.620
**MDSS compliance** (ref: yes)					
no	0.86 (0.56-1.31); 0.478	0.83 (0.55-1.23); 0.345	1.30 (0.85-2.00); 0.229	0.81 (0.58-1.12); 0.204	0.83 (0.61-1.13); 0.231
**Nut consumption** (ref: infrequently or never)	Overall *p* = ~~P=~~0.350	Overall *p* = ~~P=~~0.470	Overall *p* = ~~P=~~0.142	Overall *p* = ~~P=~~0.303	-
daily	1.71 (0.68-4.27); 0.253	1.02 (0.48-2.20); 0.950	1.37 (0.64-2.89); 0.416	0.69 (0.34-1.40); 0.308	0.65 (0.35-1.22); 0.183
weekly	1.79 (0.84-3.65); 0.136	1.76 (0.87-3.55); 0.115	0.65 (0.33-1.29); 0.222	0.62 (0.35-1.11); 0.107	0.80 (0.48-1.32); 0.379
monthly	1.21 (0.74-1.97); 0.445	1.11 (0.70-1.76); 0.660	0.64 (0.38-1.06); 0.083	1.03 (0.70-1.52); 0.878	0.58 (0.39-0.84); 0.004
**Physical activity** (ref: intensive)	Overall *p* = ~~P=~~0.689	Overall *p* = ~~P=~~0.610	Overall ~~P=~~ *p* = 0.517	Overall *p* = ~~P=~~0.207	-

Formatted: MDPI_3.1_text
Formatted: Font: Italic
Formatted: Left
Formatted: Font: Italic
Formatted: Not Superscript/ Subscript
Formatted: Not Superscript/ Subscript
Formatted: Not Superscript/ Subscript
Formatted: Not Superscript/ Subscript

light	1.00 (0.47-2.13); 0.996	0.70 (0.34-1.43); 0.326	0.98 (0.46-2.10); 0.958	1.52 (0.84-2.73); 0.166	1.10 (0.64-1.88); 0.725
moderate	0.83 (0.42-1.64); 0.596	0.79 (0.41-1.53); 0.486	0.77 (0.38-1.56); 0.474	1.12 (0.66-1.91); 0.672	0.64 (0.40-1.04); 0.074
**WHtR** (≥ 0.5, ref: yes)					
no	0.47 (0.21-1.05); 0.066	0.32 (0.15-0.66); 0.002	0.33 (0.09-1.15); 0.081	0.37 (0.14-0.99); 0.049	0.96 (0.49-1.90); 0.913
**Hypertension** (systolic ≥140 mmHg or diastolic ≥90 mmHg or treated for hypertension, ref: yes)					
no	0.76 (0.51-1.14); 0.184	0.83 (0.57-1.22); 0.348	0.93 (0.61-1.41); 0.717	0.72 (0.52-1.00); 0.051	1.31 (0.97-1.77); 0.082
**Diabetes** (≥7 mmol/L or treated for diabetes type 2, ref: yes)					
no	1.89 (1.20-2.97); 0.006	1.63 (1.05-2.54); 0.030	0.56 (0.35-0.90); 0.017	0.87 (0.59-1.29); 0.493	1.01 (0.69-1.48); 0.951
**Gout** (uric acid ≥404 µmol/L♂, ≥338 µmol/L♀ or gout, ref: yes)					
no	1.47 (0.97-2.24); 0.073	1.22 (0.82-1.84); 0.328	0.64 (0.41-0.99); 0.045	0.43 (0.31-0.60); <0.001	0.90 (0.65-1.26); 0.541

MDSS - Mediterranean Diet Serving Score. WHtR - waist-to-height ratio. ♂: males. ♀: woman. Multivariate logistic regression models were built separately for cholesterol, LDL, HDL and triglycerides. Multivariate ordinal regression model was used for the analysis of the characteristics associated with elevated fibrinogen. Adjusted odds ratios, 95% confidence intervals and *p*-Values were calculated using multivariate regression models; each of the five models presented here was simultaneously adjusted for all the covariates listed in this table.

Table S3. Characteristics associated with hypertension, diabetes, metabolic syndrome and gout as determined by the multivariate logistic regression analyses (sample size is 897 ~~participants~~ subjects without previous cardiovascular disease ~~CVD~~ diagnosis and older than 65 years of age; all independent variables included in the model are listed in the table).

	**Hypertension (systolic ≥140 mmHg or diastolic ≥90 mmHg or treated for hypertension)** Adjusted odds ratio (95% confidence interval); *p*-Value~~P~~	**Diabetes (≥7 mmol/L or treated for diabetes type 2)** Adjusted odds ratio (95% confidence interval); *p*-Value~~P~~	**Elevated HbA1c (≥6.5 mmol/L or treated for diabetes type 2)** Adjusted odds ratio (95% confidence interval); *p*-Value~~P~~	**Metabolic syndrome** Adjusted odds ratio (95% confidence interval); *p*-Value~~P~~	**Gout (uric acid ≥404 mmol/L♂-≥338 mmol/L♀ or gout)** Adjusted odds ratio (95% confidence interval); *p*-Value~~P~~
**Sex** (referent (ref): female)					
male	1.29 (0.90-1.86); 0.171	1.99 (1.24-3.19); 0.005	1.33 (0.80-2.19); 0.268	0.36 (0.24-0.53); <0.001	1.12 (0.75-1.67); 0.575
**Age (years)**	0.99 (0.97-1.03); 0.779	1.02 (0.98-1.06); 0.268	1.04 (1.00-1.08); 0.038	0.97 (0.94-1.00); 0.069	1.05 (1.01-1.08); 0.006
**Place of residence** (ref: Split)					
Island of Vis	0.59 (0.36-0.95); 0.032	0.98 (0.50-1.92); 0.949	0.97 (0.46-2.07); 0.940	2.37 (1.35-4.17); 0.003	1.75 (0.98-3.14); 0.060
Island of Korčula	1.07 (0.70-1.63); 0.771	1.38 (0.75-2.55); 0.296	1.91 (0.97-3.77); 0.061	0.84 (0.53-1.33); 0.454	1.66 (0.98-2.80); 0.061
**Education** (years of schooling, ref: ≥13)	Overall *p* = ~~P=~~0.007	Overall *p* = ~~P=~~0.150	Overall *p* = ~~P=~~0.417	Overall *p* = ~~P=~~0.653	Overall *p* = ~~P=~~0.020
0-8	1.62 (1.03-2.57); 0.038	1.93 (0.99-3.73); 0.052	1.60 (0.80-3.22); 0.186	1.20 (0.73-1.99); 0.466	1.16 (0.69-1.93); 0.579
9-12	0.93 (0.60-1.44); 0.749	1.67 (0.88-3.20); 0.119	1.41 (0.71-2.80); 0.330	1.02 (0.63-1.64); 0.948	0.66 (0.40-1.09); 0.106

**Material status** (ref: 4th quartile)	Overall *p* = P=0.497	Overall *p* = P=0.031	Overall *p* = P=0.092	Overall *p* = P=0.444	Overall *p* = P=0.581
1st quartile	0.81 (0.50-1.32); 0.405	1.79 (0.88-3.64); 0.111	1.41 (0.68-2.93); 0.350	0.74 (0.43-1.25); 0.260	0.70 (0.41-1.17); 0.173
2nd quartile	0.88 (0.54-1.44); 0.618	1.71 (0.83-3.53); 0.147	1.23 (0.58-2.60); 0.596	1.00 (0.58-1.73); 0.992	0.74 (0.44-1.26); 0.264
3rd quartile	0.68 (0.41-1.15); 0.148	2.84 (1.37-5.87); 0.005	2.24 (1.06-4.73); 0.034	0.96 (0.54-1.71); 0.891	0.81 (0.46-1.41); 0.452
**Smoking** (ref: never-smokers)	Overall *p* = P=0.210	Overall *p* = P=0.166	Overall *p* = P=0.347	Overall *p* = P=0.549	Overall *p* = P=0.005
current smokers	1.11 (0.69-1.79); 0.660	0.61 (0.30-1.24); 0.173	0.76 (0.36-1.62); 0.481	0.83 (0.48-1.43); 0.502	0.69 (0.38-1.24); 0.210
ex-smokers	0.75 (0.52-1.08); 0.121	1.26 (0.80-1.99); 0.324	1.32 (0.80-2.17); 0.274	0.81 (0.54-1.22); 0.315	1.69 (1.15-2.51); 0.008
**Alcohol intake** (ref: none)	Overall *p* = P=0.474	Overall *p* = P=0.473	Overall *p* = P=0.496	Overall *p* = P=0.009	Overall *p* = P=0.220
excessive	0.73 (0.44-1.21); 0.228	0.79 (0.42-1.46); 0.448	0.76 (0.39-1.48); 0.422	1.59 (0.90-2.82); 0.109	1.06 (0.62-1.82); 0.837
moderate	0.92 (0.65-1.30); 0.639	0.76 (0.48-1.19); 0.224	0.76 (0.47-1.21); 0.248	0.76 (0.51-1.14); 0.187	1.36 (0.93-1.99); 0.115
**MDSS compliance** (ref: yes)					
no	1.34 (0.99-1.83); 0.062	0.59 (0.40-0.87); 0.008	0.60 (0.40-0.91); 0.015	1.14 (0.80-1.62); 0.458	1.63 (1.16-2.31); 0.005
**Nut consumption** (ref: infrequently or never)	Overall *p* = P=0.496	Overall *p* = P=0.583	Overall *p* = P=0.051	Overall *p* = P=0.659	Overall *p* = P=0.968
daily	0.97 (0.53-1.78); 0.921	0.61 (0.26-1.43); 0.253	0.29 (0.10-0.87); 0.028	0.84 (0.43-1.64); 0.607	0.89 (0.44-1.82); 0.757
weekly	1.18 (0.72-1.96); 0.509	0.88 (0.45-1.72); 0.704	0.71 (0.34-1.47); 0.360	0.75 (0.43-1.30); 0.306	0.99 (0.57-1.72); 0.968
monthly	1.32 (0.91-1.91); 0.148	0.79 (0.49-1.28); 0.337	0.61 (0.36-1.01); 0.056	0.82 (0.55-1.23); 0.345	0.91 (0.61-1.37); 0.660
**Physical activity** (ref: intensive)	Overall *p* = P=0.770	Overall *p* = P=0.291	Overall *p* = P=0.336	Overall *p* = P=0.484	Overall *p* = P=0.112
light	0.97 (0.56-1.70); 0.927	1.69 (0.84-3.39); 0.141	1.68 (0.79-3.56); 0.176	0.85 (0.45-1.62); 0.632	0.95 (0.52-1.73); 0.863
moderate	0.88 (0.53-1.44); 0.605	1.31 (0.69-2.46); 0.410	1.29 (0.65-2.56); 0.470	0.73 (0.41-1.30); 0.291	1.38 (0.81-2.36); 0.236
**WHtR** (≥ 0.5, ref: yes)					
no	0.61 (0.31-1.21); 0.158	1.26 (0.41-3.89); 0.692	0.68 (0.15-3.05); 0.611	0.17 (0.08-0.37); <0.001	0.75 (0.30-1.92); 0.554
**Hypertension** (systolic ≥140 mmHg or diastolic ≥90 mmHg or treated for hypertension, ref: yes)					
no	nNa	0.67 (0.45-1.01); 0.054	0.64 (0.42-0.99); 0.046	0.48 (0.34-0.67); <0.001	0.50 (0.36-0.71); <0.001
**Diabetes** (≥7 mmol/L or treated for diabetes type 2, ref: yes)					
no	0.68 (0.46-1.02); 0.064	na	na	0.21 (0.12-0.36); <0.001	0.85 (0.57-1.27); 0.436
**Metabolic syndrome** (ref: yes)					
no	0.47 (0.33-0.65); <0.001	0.20 (0.11-0.36); <0.001	0.22 (0.12-0.41); <0.001	na	0.56 (0.38-0.84); 0.005
**Gout** (uric acid ≥404 µmol/L♂-≥338 µmol/L♀ or gout); yes is referent					

Formatted: Not Superscript/ Subscript

Formatted: Not Superscript/ Subscript

Formatted: Not Superscript/ Subscript

Formatted: Not Superscript/ Subscript

Formatted Table

no	0.50 (0.36-0.70); <0.001	0.84 (0.56-1.26); 0.399	0.92 (0.60-1.41); 0.694	0.54 (0.36-0.81); 0.003	na

MDSS - Mediterranean Diet Serving Score. WHtR – waist-to-height ratio. na – not applicable. ♂: -males. ♀: woman.

Multivariate logistic regression models were built separately for hypertension, diabetes, metabolic syndrome and gout. Adjusted odds ratios, 95% confidence intervals and *p*-VP values were calculated using multivariate logistic regression; each of the four models was simultaneously adjusted for all covariates listed in this table, with an exception of excluding predictor variables in models where those variables were the outcome variables (marked with ‘’na’’).

Formatted: Font: 9 pt, Not Italic, Font color: Black

Formatted: Space After: 6 pt, Line spacing: At least 13 pt

Formatted: Font: 9 pt, Not Italic, Font color: Black

Formatted: Font: 9 pt, Font color: Black

Formatted: Font: 9 pt, Not Italic, Font color: Black

Formatted: Font: 9 pt, Not Italic, Font color: Black

Formatted: MDPI_4.3_table_footer
